# Prevalence and factors associated with diabetes mellitus and impaired fasting glucose level among members of federal police commission residing in Addis Ababa, Ethiopia

**DOI:** 10.1186/s12902-016-0150-6

**Published:** 2016-11-28

**Authors:** Tariku Tesfaye, Bilal Shikur, Tariku Shimels, Naod Firdu

**Affiliations:** 1 Ethiopian Police University College, Police Health Professionals Training Institute, Addis Ababa, Ethiopia; 2 Addis Ababa University, School of Public Health, Addis Ababa, Ethiopia; 3 Ethiopian Federal Police Commission Health Service Directorate, Medical Logistics and Pharmaceutical Service Coordination, Addis Ababa, Ethiopia

**Keywords:** Associated factors, Diabetes mellitus, Federal Police Commission, Impaired fasting glucose, Impaired glucose homeostasis, Prevalence

## Abstract

**Background:**

The prevalence of diabetes mellitus and factors associated with it, nowadays, are increasing in alarming rates among different occupational groups. Of these occupational groups are Police officers that, often, are exposed to unique life styles and stressful situations which may lead to diabetes mellitus and other cardiovascular diseases. Due to this reason, the present study was conducted to assess the prevalence and factors associated with diabetes mellitus and impaired fasting glucose level among members of federal police commission residing in Addis Ababa, Ethiopia.

**Methods:**

A cross-sectional study design was conducted from April to May 2015. Multistage and systematic random sampling techniques were employed to select the study participants. The study population was federal police commission members living in Addis Ababa and served for at least a year. The data were collected using structured questionnaire, physical examinations and blood samples, based on the WHO stepwise approach. Data were entered in to SPSS version 20.0 and descriptive statistics and logistics regression were used for analysis.

**Results:**

Out of the 1003 eligible subjects, 936 (93.3%) police officers have participated in this study. The prevalence of overall impaired glucose homeostasis (IGH) was 120 (13%) of which 47 (5%) were diabetes and 73 (8%) were impaired fasting glucose. Whereas police rank, history of first degree relative who suffered from diabetes, hypertension and waist hip ratio showed a statistical significance with prevalence of diabetes mellitus, age, family history, hypertension, BMI and waist hip ratio were found to be associated with impaired fasting glucose.

**Conclusion:**

The study identified a high prevalence of IGH among the police officers. A priority should be given on preventive strategies of diabetes mellitus, as that of communicable diseases, by Federal Police Commission Health Service Directorate, Federal Ministry of Health and other concerned partners.

## Background

Diabetes mellitus is characterized by chronic hyperglycemia which becomes an emerging public health problem due to its high prevalence, association with cardiovascular diseases, and overall morbidity and mortality [1]. A recent estimate indicates that more than 387 million (8.5%) of people worldwide have diabetes. Among these Africa account a 22 million (5.1%) people with diabetes which is likely to increase by 70% in 2035 [2].

Among people with diabetes mellitus in developing countries, the majority were in the age group of 45 to 64 years while those in developed countries are aged 65 years and above. This indicates that developing countries are losing productive age groups than developed nations. This, in turn, brings double burden in the region; along with the communicable diseases such as; HIV/AIDS, tuberculosis and other infectious diseases [3].

Before the 1990s, diabetes mellitus was considered as a rare medical condition in Sub-Saharan Africa [4]. Currently, however, many studies revealed that the prevalence and incidence of type 2 diabetes is rising in the region, mostly due to life style changes (westernization), lack of physical activities, increased rural urban migration (urbanization), high calorie intake and increased life expectancy (ageing) of the population [5, 6].

According to the World Health Organization, in Sub- Saharan Africa, the number of diabetes cases in 2013 ranges from 4.5 to 5.0% [6]. This could increase by 98% which is from 12.1 million in 2010 to about 23.1 million in 2030. The impaired glucose tolerance that was reported in 2010 (26.9 million) is also expected to rise to 47.3 million in 2030 [7].

In Ethiopia, it is difficult to find population based data on the exact prevalence of diabetes. However, there are some studies done on selected population groups that showed a prevalence of 4.6 to 5.1% diabetes [8–10]. It is also the second cause for patients to attend for health care service in hospitals of the country [11]. According to the 2014 report of the International Diabetes Federation (IDF), the number of people aged 20–79 years and living with diabetes in Ethiopia was estimated to be 4.9 million and more than 2.9 million (6.9%) people live with impaired glucose tolerance. Among these, more than 1.4 million people were undiagnosed for diabetes mellitus and its prevalence is higher in urban than rural population [2].

Globally, the prevalence of diabetes across various occupational groups and its relationship with an occupational factor is a topic of recent interest. Police officers as an occupational group are exposed to unique life styles and stressful situations that may lead to diabetes mellitus and other cardiovascular diseases. For instance, the study conducted among police officer in India showed that this occupational group had higher prevalence of metabolic syndrome and other cardio metabolic abnormalities compared to the general population [12, 13]. In US, a cohort study conducted among police force members also showed that these groups are at risk of developing non communicable diseases at earlier age and that they die much earlier compared to other groups [14]. But the situation of Ethiopian police officers is unknown, even though the groups are predisposed to those factors accountable for developing diabetes mellitus.

Evidence from many studies indicates that police officers with well managed diabetes mellitus are capable of safe and effective job performance on duty [12–15]. However, those who are undiagnosed may be at risk of sudden incapacitation and death, thus affecting their wellbeing and ability to perform well. Knowing the prevalence of diabetes mellitus and impaired fasting glucose level and associated factors among police officers will give us a clue for appropriate intervention.

Therefore, the present study was conducted to estimate the prevalence of diabetes mellitus and impaired fasting glucose level and to identify possible associated factors among members of Federal Police Commission in Addis Ababa, Ethiopia.

## Methods

### Study area

The study was conducted in Addis Ababa city, the capital of Ethiopia, with a population of 3,048,631 and total area of 540 km^2^. It is a metropolitan city currently structured in ten sub-municipalities (KifleKetemas) and 203 districts (Woredas) [16].

Federal police, which is the interest of this study, is governed under the accountability of the prime minister and is structured as Ethiopian Federal Police Commission. The Commission has four main sectors (zerf) namely; Federal police commission human resource development and technology expansion, Federal police commission crime prevention, Federal police commission crime investigation and Ethiopian police University College sectors. Within each sector, there are departments. This study used the structures and departments that were found in Federal Police Commission [17].

### Study design and period

The study used a community based cross-sectional study design conducted from April to May 2015.

### Source population

All Ethiopian Federal police commission officers who live and work in Addis Ababa.

### Study population

Federal Police commission officers who live in Addis Ababa and fulfilled the inclusion criteria.

### Sample size

Sample size was determined by using the single proportion formula and prevalence of diabetes mellitus that was reported in Bishoftu town, Ethiopia [9].

$$ \mathbf{n}=\frac{{\left(\mathbf{Z}\alpha /2\right)}^2\mathbf{p}\left(\mathbf{1}\mathbf{\hbox{-}}\mathbf{p}\right)}{{\mathrm{d}}^2} $$

where;

α = 0.05 or Zα/2 = 1.96

*p* = 0.05, from study conducted in Bishoftu town

*d* = 0.02

$$ \mathrm{n}=\frac{(1.96)^20.05\left(1-0.05\right)}{0.02^2}=456 $$

By using the design effect (multiplying by 2), and adding 10% non-response rate, the final sample size was; 456x2 = 912+ (912x10%) =1,003 subjects.

### Sampling procedures

A multi-stage sampling technique was employed in order to select a representative sample of respondents from the study population. At first, a list of all main sectors along with the respective departments was sorted out from which two departments were selected randomly. The total sample size was, then, distributed proportionally to the population size of each department. Lastly, respondents were selected using a systematic random sampling from the sampling frame.

### Data collection procedure

A 4-days training on interviewing technique, questionnaire administration and physical measurement techniques, were given to the data collectors a week before the actual survey. Data was collected by two nurses, two health officers and two-laboratory technicians.

Participant’s eligibility was determined by verifying the time of their last meal in order to ascertain that they have undergone an overnight fasting of at least eight hours. Socio-demographic data and relevant behavioral and life style characteristics were recorded in pre-tested questionnaires.

Participants were informed about the purpose of the study through the data collectors to enhance maximum participation. Those who had agreed to participate were requested to be registered at the office and were informed when to undergo an overnight fasting before their test. Then, anthropometric measurements and biochemical tests were taken and advice was recommended for each participant.

### Definition of variables

**Police officer**: an individual who had taken at least 6 months of police training and working within the Federal Police Commission

**Rank**: A designation given to police officers based on service year, professional qualification in education or work efficiency

**Lower rank**: a category of the ranks from ‘Constable’ to ‘Chief Sergeant’

**Middle rank**: a category of the ranks from ‘Assistant Inspector’ to ‘Inspector’

**Higher rank**: a category of the ranks from ‘Chief Inspector’ to ‘General Commissioner’

**First degree relative who suffered from diabetes and/hypertension:** a previous history of the respondent’s father, mother, full brother or sister had diabetes and/ hypertension.

**Current smoker:** an individual who reported smoking during the time of the study

**Current drinker**: an individual who were alcohol drinker during the time of study

**Low consumption of vegetable and fruits**: an individual who do not consume fruits and vegetables daily

**Diabetes mellitus**: a fasting capillary whole blood glucose value equal to or greater than 6.1 mmol/l (≥110 mg/dl)

**Hypertension**: the average systolic blood pressure readings ≥140 mmHg and/or diastolic blood pressure readings ≥90 mmHg

**Impaired fasting glucose**: a fasting capillary whole blood glucose value ≥5.6 mmol/l (≥100 mg/dl) and <6.1 mmol/l (110 mg/dl)

**Vigorous physical activity**: an individual who reports 1500 MET minutes per week

**Moderate physical activity**: an individual who reports 600 to 1500 MET minutes per week

**Poor Physical activity**: an individual who reports <600 MET minutes per week

### Data quality management

The study used a modified form of the WHO Global Risk Factor Surveillance Questionnaire [18]. Pretest was carried out to ensure suitability of the questionnaires for the survey. An Amharic translated version of the questionnaire was used during the study. Physical measurements were recorded twice and in some case three times in order to minimize observer error in measurements and records, whereas, rotation of data collectors was done to compare values. The sphygmomanometer used in the field was compared with the one normally used in Federal police referral hospital before and after data collection each day. The glucometer device and strips were checked periodically for consistency in reference and test reading. Cleaning, Coding and recording of the data collected was done each day.

### Data analysis

Data were entered into SPSS version 20.0, cleaned manually and analyzed. Frequency distributions and percentage tables were used to present socio-demographic variables and behavioral characteristics. Prevalence of diabetes mellitus and impaired fasting glucose was showed in percentages. Cross tabulation, and 95% confidence interval was used to present results of bivariate analysis. Multivariate logistic regression analyses were done to control potential confounding variables and determine factors associated to presence of diabetes mellitus and impaired fasting glucose.

## Results

Out of the 1003 eligible subjects, a total of 936 (93.3%) police officers aged from 18 to 55 years have participated in this study. Sixty (6%) subjects were not available due to work related problems and 7 (0.7%) were on annual leave during the study period.

### Socio-demographic characteristics

Among the total respondents, 740 (79.1%) were male and 196 (20.9%) were female, with a male to female ratio of 3.8:1. The age of the study participants ranged from 18 to 55 years, with a mean and median age of 29.53 ± 8.722 and 27.0 years respectively. The interquartile range for age was between 23 and 34 years. Majority of the respondents were lower rank police officers which constituted 710 (75.8%), followed by middle rank accounting of 125 (13.4%) subjects.

Five hundred seventy eight (61.8%) of the study subjects served the police commission below 10 years, followed by 203 (21.7%) and 155 (16.6%) who served from 11 to 20 years and greater than 20 years respectively. Nearly two third (66.3%) of the respondents belong to the orthodox Christian religion.

More than half (56%) of the study participants were single. However, there were also a high proportion (39.5%) married respondents. The rest 42 (4.5%) were divorced, separated or widowed. Fifty six (6.0%) of the study subjects have first degree relative who suffered from diabetes mellitus (Table 1).

**Table 1:** Socio-demographic characteristics of study participants in Federal Police Commission; May 2015, Addis Ababa, Ethiopia (*n* = 936)

Characteristics	Number	Percent
Gender		
Male	740	79.1
Female	196	20.9
Age		
< 25	342	36.6
25–34	362	38.7
35–44	142	15.2
≥ 45	89	9.5
Police Rank		
Lower rank	710	75.8
Middle rank	125	13.4
Higher rank	101	10.8
Years of service		
1–10	578	61.7
11–20	203	21.7
> 20	155	16.6
Religion		
Orthodox Christian	625	66.3
Muslim	121	12.9
Protestant	183	19.6
Other	11	1.2
Marital status		
Single	524	56.0
Married	370	39.5
Others	42	4.5
History of 1^st^ degree relative suffered from diabetes		
Yes	56	6.0
No	795	84.9
Education level		
Primary school	15	1.6
Secondary school	659	70.4
College & University	262	28.0
Income level^a^		
< 1500	311	33.2
1500–2199	302	32.3
2200–3199	160	17.1
≥ 3200	163	17.4

The variable ‘history of 1st degree relative' is seemingly not adding to 100%, as the subjects responded as 'do not know' were excluded both from socio-demographic [this table] and from the consequitive analyses

^a^Measure is in Ethiopian Birr [ETB]

### Behavioral characteristics of the study participants

Overall, 63 (6.7%) of the study participants reported that they had history of smoking cigarettes in their life time, out of which 47 (5%) were current smokers. Among current smokers, majority (83%) were daily smokers. Approximately 55% of the current smokers just smoked cigarettes for 10 or more years and the number of cigarettes smoked ranged from 3 to 15 sticks per day, with a mean of 7 cigarettes per day. The numbers of participants who have ever chewed khat (stimulant leaf) were 104 (11.1%).

Among the study subjects, 664 (71%) had ever consumed alcohol in their life of which 656 (98.5%) were current drinkers. Of those who ever consumed alcohol, 45.1% drink alcohol less than 3 days a month, followed by 162 (24.6%) from 1 to 4 days per week. Majority (88.2%) of the study participants do not eat vegetables and fruits every day.

Overall, 338 (36.1%) of the study participants were inactive towards physical activity. The prevalence of physical inactivity increased with age from 18% among 18–24 years to 77.5% among those aged ≥45 years (Table 2).

**Table 2:** Behavioral characteristics of study subjects in Federal Police Commission; May 2015, Addis Ababa, Ethiopia (*n* = 936)

Characteristics	Number	Percent
Ever smoker		
Yes	63	6.7
No	873	93.3
Current smoker		
Yes	47	74.6
No	16	25.4
Chew Khat		
Yes	104	11.1
No	832	88.9
Ever taken alcohol		
Yes	664	70.9
No	272	29.1
Low consumption of fruits & Vegetables		
Yes	854	91.2
No	82	8.8
Physical activity		
Vigorous	420	44.9
Moderate	171	18.2
Poor	345	36.9

Overall, 753 (80.5%) of the study participants had body mass index (BMI) < 25 kg/m^2^. Out of the rest 183 (19.5%) study subjects who had BMI ≥25.0 kg/m^2^, 168 (91.9%) were overweight and 15 (8.1%) were obese.

### Prevalence of diabetes mellitus and impaired fasting glucose

Of the total participants who were tested, 47 (5%) had diabetes, while 75 (8%) had impaired fasting glucose with an overall impaired glucose homeostasis of 122 (13%). Out of the study participant with diabetes, 15 (31.9%) were already known diabetics whereas the rest 32 (68.1%) did not know their blood glucose status making the ratio of diagnosed to undiagnosed diabetes 1:2.1.

### Multivariate analysis of factors associated with diabetes mellitus

From the multivariate logistic regression analysis, it was shown that study participants of the higher rank were found to be 3.8 times at increased risk of having diabetes mellitus than participants with lower rank, (AOR = 3.8, 95% CI, 1.1–13.7). Having history of first degree relative who suffered from diabetes were found to be 6.9 times more likely to have diabetes mellitus (AOR = 6.9, 95% CI; 2.0–23.5). Age and years of service did not show any significant association with the prevalence of diabetes. There was no statistical significance found among those participants who ever smoked cigarettes.

Study participants with hypertension were found to be 6.7 times at risk of having diabetes mellitus than the participants with normal blood pressure (AOR = 6.7, 95% CI, 2.6–17.2). Waist hip ratio were found to have a statistical significance with prevalence of diabetes (AOR = 4.6, 95% CI, 1.9–10.9). No statistical significance was also found on BMI of the study participants (Table 3).

**Table 3:** Multivariate associations of socio-demographic, behavioral, and anthropometric measurement characteristics with diabetes mellitus among study participants in Federal Police Commission; May 2015, Addis Ababa, Ethiopia (*n* = 936)

Presence of diabetes mellitus				
Characteristics	Yes (%)	N (%)	COR	AOR
Age				
< 25	2 (0.6)	341 (99.4)	1.0	1.0
25–34	6 (1.7)	356 (98.3)	46.3 (10.5–203)	2.9 (0.2–36.1)
35–44	20 (14.1)	122 (85.9)	16.1 (6.2–41.8)	4.4 (0.7–26.6)
≥ 45	19 (21.3)	70 (78.7)	1.7 (0.8–3.3)	0.8 (0.3–2.3)
Police rank				
Lower rank	14 (2.0)	669 (98.0)	1.0	1.0
Middle rank	8 (6.4)	117 (93.6)	16.4 (8.2–32.8)	4.6 (1.3–16.1)
Higher rank	25 (24.8)	76 (75.2)	4.8 (2.1–11.2)	3.8 (1.1–13.7)
Years of service				
1–10	5 (0.8)	626 (99.2)	1.0	1.0
11–20	14 (8.0)	161 (92)	34.4 (13.0–91.1)	1.2 (0.2–8.9)
> 20	28 (21.5)	102 (78.5)	3.2 (1.6–6.3)	0.6 (0.2–2.1)
Family history				
Yes	11 (19.6)	45 (80.4)	5.6 (2.7–11.9)	6.9 (2.0–23.5)
No	33 (4.2)	762 (95.8)	1.0	1.0
Ever Smoked				
Yes	11 (17.5)	52 (82.5)	4.9 (2.4–10.2)	0.8 (0.3–2.4)
No	36 (4.1)	837 (95.6)	1.0	1.0
Hypertension				
Yes	11 (1.4)	758 (98.8)	19.0 (9.4–38.4)	6.7 (2.6–17.2)
No	36 (21.6)	131 (78.4)	1.0	1.0
BMI				
< 25	13 (1.7)	740 (98.3)	1.0	1.0
25–29.9	28 (16.7)	140 (83.3)	38.0 (11.8–122.2)	1.7 (0.3–9.4)
≥ 30	6 (40.6)	9 (60.4)	3.3 (1.1–10.1)	0.7 (0.2–3.4)
WHR				
Obese	30 (18.0)	137 (82.0)	9.7 (5.2–18.1)	4.6 (1.9–10.9)
Normal	17 (2.2)	752 (97.8)	1.0	1.0

*p* < 0.05

### Multivariate analysis of factors associated with IFG

In binary logistic regression analysis, the study participants aged ≥45 years were found to be 4.9 times at risk of having impaired fasting glucose compared to those <25 years of age (AOR = 4.9 95% CI, 1.04–23.1). A family history of diabetes has also showed an independent significant association with the prevalence of IFG. Participants with positive family history of diabetes were about 3 times more likely to develop IFG than study subjects with no family history of diabetes (AOR = 3.2 95% CI, 1.4–7.5). Police rank and years of service did not show a significant association with the prevalence impaired fasting glucose level too.

Hypertension (both systolic and diastolic hypertension), body mass index and waist hip ratio were found to have statistical significance with IFG. Study subjects with hypertension were found to be 4.5 times at risk of having IFG than study subjects with normal blood pressure (AOR = 4.5, 95% CI, 2.6–7.8). Participants with BMI of ≥30 kg/m^2^ were found to be 6 times at risk of developing IFG than those subjects with BMI of <25 (AOR = 6.0, 95% CI, 1.2–30.2).

None of the behavioral characteristics of the study subjects were found to show statistically significant association with IFG (Table 4).

**Table 4:** Multivariate associations of socio-demographic, behavioral, and anthropometrics measurements with IFG in Federal Police Commission; May 2015, Addis Ababa, Ethiopia (*n* = 936)

Presence of IFG				
Characteristics	Yes (%)	N (%)	COR	AOR
Age				
< 25	7 (2.0)	336 (98.0)	1.0	1.0
25–34	28 (7.7)	334 (92.3)	4.0 (1.7–9.3)	1.8 (0.7–4.8)
35–44	39 (27.5)	103 (72.5)	18.2 (7.9–41.9)	3.1 (0.8–12.0)
≥ 45	46 (51.8)	43 (48.3)	51.4 (21.8–120.9)	4.9 (1.1–23.1)
Police rank				
Lower rank	47 (6.6)	663 (93.4)	1.0	1.0
Middle rank	23 (18.4)	102 (81.6)	3.2 (1.9–5.5)	0.5 (0.2–1.1)
Higher rank	50 (49.5)	51 (50.5)	13.8 (8.5–22.6)	0.9 (0.9–2.6)
Years of service				
1–10	20 (7.0)	268 (93.0)	1.0	1.0
11–20	27 (13.3)	254 (86.7)	4.3 (2.3–14.3)	2.4 (1.0–6.1)
> 20	73 (47.1)	82 (52.9)	24.8 (14.4–42.9)	3.3 (0.9–11.9)
Family history				
Yes	31 (47.0)	35 (53.0)	5.8 ((2.3–7.8)	3.2 (1.4–7.5)
No	89 (11.5)	704 (88.5)	1.0	1.0
Ever Smoked				
Yes	20 (31.7)	43 (68.3)	3.6 (2.0–6.4)	1.0 (0.4–1.4)
No	100 (11.5)	773 (88.7)	1.0	1.0
Chewing Khat				
Yes	5 (41.7)	7 (58.3)	5.6 (1.8–18.0)	2.8 (0.6–17.8)
Yes, daily	21 (22.8)	71 (77.2)	2.3 (1.4–4.0)	2.5 (1.0–6.4)
No, at all	94 (11.3)	738 (88.7)	1.0	1.0
Current alcohol consumption				
Yes	91 (13.9)	565 (86.1)	1.5 (0.2–11.6)	0.8 (0.4–1.4)
No	1 (10.0)	9 (90.0)	1.0	1.0
Physical activity				
Vigorous	20 (4.8)	401 (95.2)	1.0	1.0
Moderate	18 (10.2)	159 (89.8)	2.3 (1.2–4.4)	1.5 (0.7–3.0)
Poor	82 (24.3)	256 (75.7)	6.4 (3.8–10.7)	1.6 (0.7–3.7)
Hypertension				
Yes	76 (45.5)	91 (54.5)	13.8 (8.9–21–2)	4.5 (2.6–7.8)
No	44 (5.7)	725 (94.3)	1.0	1.0
BMI				
< 25	46 (6.1)	707 (93.90	1.0	1.0
25–29.9	64 (38.1)	104 (61.9)	9.5 (6.2–14.6)	3.0 (1.7–5.4)
≥ 30	10 (66.7)	5 (33.3)	30.7 (10.1–93.7)	6.0 (1.2–30.2)
WHR				
Obese	54 (32.3)	113 (67.7)	5.1 (3.4–7.7)	4.5 (2.6–7.8)
Normal	66 (8.6)	703 (91.4)	1.0	1.0

## Discussion

The prevalence of overall impaired glucose homeostasis, (IGH) in the present study, was 13%. Out of this, 5% were diabetes mellitus and 8% were impaired fasting glucose. Among the subjects with diabetes mellitus, the ratio of those diagnosed to the undiagnosed was 1:2.1. Sixty eight percent of the study subjects with diabetes were unaware of their blood glucose level before the survey. This higher prevalence of undiagnosed diabetes might be due to the priority given to communicable diseases, poor culture of visiting health facility for medical checkups, and lack of decentralized health services for chronic non communicable diseases. This is also similar to other cross sectional studies in developing countries that reported a higher prevalence of undiagnosed diabetes mellitus due to lack of health facility and awareness as contributing factors [19–22].

The prevalence of diabetes mellitus in the present study (5%) was consistent to the estimated national prevalence of Ethiopia (4.85%) reported by the IDF in 2014 [2]. Comparable prevalence (4.5%) was also reported by ACIPH and MIRT among commercial bank workers and teachers in Addis Ababa [10]. There was a similar study conducted in Bishoftu town which reported a 5.1% undiagnosed diabetes [9]. In contrast to this study, however, higher prevalence of 33.6, 32.1 and 15.0% of diabetes were reported by J. Ramakrishnan et al. [13], Shaban et al. [23] and Kumar et al. [15], respectively among police officers in India. This might be due to the high genetic predisposition of Indian people to diabetes mellitus, low prevalence of smoking, alcohol consumption and larger proportion of younger study subjects in the present study. Likely, higher prevalence of diabetes (7%) was reported by Enang, OE, Otu AA, Essien OE, et al. in Calabar [24]. Nevertheless, a low prevalence (6.67%) of overall IGH was reported by Manjunath et al. among army members in India [12].

The prevalence of impaired fasting glucose (8%) in this study was comparable to a finding by J. Ramakrishnan et al., on policemen in India that reported 7.0% [13]. There was also another study in Calabar by Enang OE, Otu AA, Essien OE, et al. which reported a 7% impaired fasting glucose level [21]. In contrary, lower prevalence (1.1%) of IGH was reported by Kumar et al. [15] among police officers in Bankura, India. This might be accounted for different criteria used for diagnoses of impaired fasting glucose level, other socioeconomic as well as behavioral characteristics.

The study found a significant increase in prevalence of diabetes mellitus and impaired fasting glucose with hypertension. This is similar to the findings reported by Shaban et al. [23], Kumar et al. [15], Manjunath et al. [12], among police officers in India and Megerssa et al. [9] in Ethiopia.

In this study, BMI of the study participants was found to be significantly associated with having of IFG. This is, also, similar to a study conducted by Nagaya et al. [14] among police officers in India which documented that the increasing higher prevalence of diabetes among police officers was due to their BMI compared to the general population. A cohort study by Mesinger et al. [25] has also revealed a significant association between BMI and diabetes. By a finding in Ethiopia, too, BMI was shown to be significantly associated with diabetes. In addition, central obesity was associated with the prevalence of diabetes in this study. The result was comparable with the study by Shaban et al. [23], Manjunath et al. [12] among police officers in India.

Age was found to have a significant association with impaired fasting glucose among the study subjects. This is similar to a finding by Manjunath M.L et al. [12], among army members in India which shown that IGH tend to increase with advanced age. This could be explained by the progressive decline in strength and endurance of musculature which, in turn, causes muscle atrophy thereby leading for impaired glucose level.

Even though association was established between diabetes mellitus and physical activity, it is difficult to prove the casual relationship as the number of physically active subjects with the disease was very low. However, it is evident that many cross sectional [21, 22, 26, 27] and cohort studies [28–32] have documented lack of physical activity as an established factor for having diabetes mellitus. On the other hand, people who were engaged in moderate to vigorous physical activity were found to be at a lower risk [12, 33]. Similarly, in a study done by Colberg et al. [34], it was stated that participation in regular physical activities improved blood glucose control, prevented or delayed type-2 diabetes, blood pressure, mortality and morbidity.

The other factor which was associated with both diabetes mellitus and impaired fasting glucose in the present study was, having first degree relatives who suffered from diabetes mellitus. This is also supported by many studies that proved family history as an independent factor that contributes for developing diabetes mellitus and impaired fasting glucose [12, 13, 35]. There was no significant sex difference observed in prevalence of diabetes mellitus and IFG in this study. This is consistent with reports in different countries, like Angola [36], Nigeria [21, 22], South Africa [27], Botswana [37], China [26] and the 3^rd^ NHANES, in USA [35]. In general, even though, some gender differences have been observed in some communities across the globe, the variation might be attributed for prevalence of different factors in different populations [29, 35, 38].

There was also no significant ethnic and religion difference on IGH among the study subjects in contrast to other global studies such as; Pima Indians, African Americans and Arabs [35]. The indifference might be due to cultural and life style similarity among the participants in the present study. Likely, there were no statistical significance differences found on police rank and years of service in this study with both diabetes mellitus and impaired fasting glucose. This is comparable with the study by Kumar et al. [15]. In contrast to this, police rank and year of service were associated with diabetes mellitus in Manjunath et al. study [12]. This might be accounted for the reason that majority of the study subjects in this survey were lower rank and served less than 10 years.

Due to resource limitation, oral glucose tolerance tests were not performed which may decrease the strength of this study. In addition, since the study was conducted among subjects with different life style and work culture, generalizing the findings for the actual population of the country would be difficult.

## Conclusion

The study identified a high prevalence of diabetes mellitus and impaired fasting glucose among the police officers. The important factors that were associated with diabetes mellitus and impaired fasting glucose were; age, physical inactivity, hypertension, BMI and central obesity. Whereas, further studies might be required to investigate the full magnitude of the problem, a priority must be given to preventive strategies of IGH by Federal Police Commission Health Service Directorate, Federal Ministry of Health and other partners.

## Abbreviations

AOR: Adjusted odds ratio
BMI: Body mass index
COR: Crude odds ratio
IFG: Impaired fasting glucose
IGH: Impaired glucose homeostasis
MET: Metabolic equivalent of task
WHO: World Health Organization
WHR: Wrist hip ratio
